# How Are Scientists Using Social Media in the Workplace?

**DOI:** 10.1371/journal.pone.0162680

**Published:** 2016-10-12

**Authors:** Kimberley Collins, David Shiffman, Jenny Rock

**Affiliations:** 1 Centre for Science Communication, University of Otago, Dunedin, New Zealand; 2 Leonard and Jayne Abess Center for Ecosystem Science and Policy, University of Miami, Miami, FL, United States of America; University of Bologna, ITALY

## Abstract

Social media has created networked communication channels that facilitate interactions and allow information to proliferate within professional academic communities as well as in informal social circumstances. A significant contemporary discussion in the field of science communication is how scientists are using (or might use) social media to communicate their research. This includes the role of social media in facilitating the exchange of knowledge internally within and among scientific communities, as well as externally for outreach to engage the public. This study investigates how a surveyed sample of 587 scientists from a variety of academic disciplines, but predominantly the academic life sciences, use social media to communicate internally and externally. Our results demonstrate that while social media usage has yet to be widely adopted, scientists in a variety of disciplines use these platforms to exchange scientific knowledge, generally via either Twitter, Facebook, LinkedIn, or blogs. Despite the low frequency of use, our work evidences that scientists perceive numerous potential advantages to using social media in the workplace. Our data provides a baseline from which to assess future trends in social media use within the science academy.

## Introduction

The rapid development of social media has changed the way people interact with one another, access and share information [1, 2, 3, 4]. While traditional forms of media are one-way in nature and disseminate messages from a single point to an intended audience, social media facilitates two-way interaction and allows information to proliferate within an electronic community [5, 6]. This potential for knowledge exchange and networking appears to be recognized within academia. In 2012, Priem et al. surveyed a range of academic scholars in the US and UK and found that 3% of them were active on Twitter; they predicted that this number would increase steadily [7]. Yet in 2013, already 23% of American adults used Twitter (with 71% using Facebook, and 52% using multiple social media platforms [8]). This disparity suggests a gap in the uptake of social media in the academic work place.

One area of academia in which it might be expected that social media might be more quickly adopted is within the sciences, due to the increasingly respected practice of science communication with the general public. Indeed, there is evidence that scientists are using social media for communicating specific aspects of their research, as well as science more generally, as a means of outreach to increase engagement and science literacy [6, 9]. It also appears that scientists will sometimes use social media for the facilitation and exchange of knowledge within and among scientific communities (communication internal to science) [5, 10]. However, the limited literature to date that investigates the frequency of scientists using social media suggests only a very slow increase in its usage. A 2013 survey of environmental scientists showed few were actively using social media in their work [11] and in fact a 2014 study of scientists at a major US university, indicated that they were explicitly not using certain social media platforms (e.g. 40% expressed that they would not use Twitter for professional or academic work [12]). What might be the perceived benefits or barriers to social media use by scientists in their work? So far the published data is patchy, and general baseline data is needed to allow us to assess trends in the uptake of different social media platforms by scientists.

This study aims to provide insight into how some scientists are using social media to create scholarly connections outside their own department, share and discuss research, and communicate with the public. We present the results of an international survey of scientists who are using social media in their workplace. Our analysis interrogates their use of different types of social media platforms including blogs, Facebook, and in particular, Twitter, as well as their general perspectives about social media use at their work.

## Methods

The University of Otago Ethics Committee granted permission for this research (reference number D13/352). We conducted a survey to assess the behaviour of scientists when using various social media platforms, and thus specifically targeted scientists who were already using social media. A survey of 52 questions was produced and distributed electronically using online survey software (SurveyMonkey, www.surveymonkey.com). Questions were designed to measure quantitative and qualitative responses, including a mix of multiple choice, short answer, and preference ranking (e.g. on a scale of “never” to “often”, or as a list with 1 being the most often). Representative quotes from an emergent thematic analysis of short answer questions have been included. The general content and scope of questions is outlined in Table 1.

**Table 1:** The content and scope of questions included in the survey. All questions in their original detail are available in SI File.

Question #	General content	Scope
3–8	Demographics	From age and nationality to degree and institutional affiliation
9–10	General use of social media	From use of specific social media services to frequency of use
11–16	Use of Facebook	From use of Facebook to interactions with pages that focus on science.
17–23	Use of Blogs	From reading science blogs to sharing and writing them.
24–52	Use of Twitter	From metrics on followers to Tweets to topics and perceived attitudes in the workplace, among peers and at conferences

### Sample Pool

Participants were recruited using a snowball sampling method initiated by an email invitation to participate. This was distributed to 515 scientists who identified themselves (as of 09/02/2013) as “scientists who use Twitter” on the Tweet Your Science website (www.tweetyourscience.com). The database was used because it was the largest online database of scientists using social media at the time. It was also advertised on Twitter (with a total of 172 retweets) for a period of 2 weeks. The survey was open from October 2, 2013 until September 19, 2014. Although the survey was distributed internationally, it was not translated and so targeted English-speaking scientists.

## Results and Discussion

### Respondent demographics

A total of 587 individuals identifying as scientists completed the survey between 02/10/2013 and 02/10/2014. Of the original 515 emailed, 203 responded (40% response rate) with the rest attributed to the ‘snowball effect’. Respondents were from 31 countries, the most common being the United States of America (37%), the United Kingdom (19%), New Zealand (14%), Australia (11%) and Canada (10%). Respondents were nearly evenly split between male (49%) and female (51%). The majority of participants identified as belonging to age brackets of 21–29 years (39%) and 30–39 years (39%). A further 14% were aged between 40–49 years, and 7% between 50–59 years, with just 1% aged 60 years or older and 0.4% (n = 2) aged 18–20. With the participants targeted as active social media users, the fact that 78% of them represented the 21–39 age bracket suggests that younger scientists may well rely more on social media channels, a finding at odds with the trends reported by some studies [13], though consistent with others [5].

More than half (54%) of survey respondents held a Doctor of Philosophy (PhD) as their highest form of education. Most of the remainder held a Masters (23%), Bachelors or Honors degree (13% and 6%, respectively), with few holding lesser degrees of postgraduate Diploma, or high school Certificate (2 and 1%, respectively). Participants who selected ‘Other’ identified as being current candidates for a postgraduate degree (n = 4) or a Doctor of Medicine (n = 2). The vast majority of survey respondents were associated with a university or college (87%), with fewer associated with a research institution or governmental department (each 5%). Less represented were hospitals (n = 5), not for profit organisations (n = 4), private industry (n = 4), museums (n = 3) and freelance scientists (n = 1).

A diversity of scientific fields were represented, with the most common being ecology (13%), biology (5%) and psychology (5%), followed by genetics, molecular biology, conservation biology, neuroscience, microbiology, chemistry and evolutionary biology (each 4%). For 13% of respondents, “other” was selected as their field of expertise, which, in several instances was used to indicate a highly specialised area not provided as an option. For example, “entomology” was specified (rather than selecting the encompassing field of “zoological sciences”). In most cases, however, “other” was selected to indicate more than one area of expertise (e.g. both ecology and molecular biology).

Notably, although our survey captured some geographic diversity across a balanced sampling of males and females, our results are very much biased towards responses from younger scientists, heavily representing the life sciences within academia.

### General use of social media and blogs

A variety of social media services were reported as being used by the scientists, but three dominated. Twitter, Facebook and LinkedIn were used by over 50% of respondents (specifically by 88%, 82% and 66%, respectively; Table 2). There were fewer users of Google+, Wordpress and Research Gate (40%, 34% and 31%, respectively), and far fewer of the others, including Instagram (21%), Pinterest (18%), Mendeley (19%), Tumblr (14%), Blogger (12%) and Reddit (13%). Services indicated by participants as “Other” included Academia.edu (n = 15), LiveJournal (n = 2), YouTube (n = 2), Flickr (n = 1), Xing (n = 1), UnTapped (n = 1) and Google Groups (n = 1), suggesting some confusion as to what defines a social media service. An individual’s understanding of this definition may vary, and is influenced by a range of factors such as exposure to the medium, level of involvement with the literature surrounding the subject, and how social media is defined in their society or more broadly in popular culture. In future, providing a definition of social media to encourage survey participants to give more refined answers would be useful.

**Table 2:** The social media services used most often by scientists. Services were ranked 1–10, with 1 being the service used most often (n = 407respondents).

Social Media Service	Average Rank	Users (n)	Non-users (n)
Twitter	1.5	512	34
Facebook	2	479	67
LinkedIn	4	399	147
Wordpress	4.5	233	313
Google +	4.9	273	272
Instagram	5.0	163	383
Research Gate	5.1	210	335
Reddit	5.4	125	421
Pinterest	5.7	141	405
Mendeley	5.8	167	379
Tumblr	6.1	127	419
Blogger	6.1	113	432
FourSquare	7.8	61	484
MySpace	8.8	44	502

Respondents were also asked to estimate their usage of different social media services. The most frequently used were Twitter, Facebook, LinkedIn, Wordpress and Google+ (all ranked < 5; Table 2). Here again there may have been room for confusion in what each participant understood as constituting being a user. For instance they may have indicated that they were a user of a service despite only being signed up for it, which should impose limitations in our interpretation of their answers. A clear definition of what being a user of a particular service entails would be usefully included in future work.

These results differ from those collected from the general public, which indicated that the social media services used most frequently were Facebook (93%), YouTube (62%), Twitter (36%), Google+ (30%), LinkedIn (22%), Pinterest (22%), MySpace (16%), Instagram (15%), Tumblr (11%), and FourSquare (6%) [14]. However, there is consistency in the relative popularity of Twitter, Facebook and Google+ (although as previously described our respondents were drawn from a database of Twitter-users).

When queried specifically about their use of blogs as a form of social media, the majority of scientists (92%) indicated that they read science blogs, and many reported they have shared blog posts with professional colleagues (84%). While only half (50%) had authored a blog themselves, the majority (89%) indicated that they believed that blogs do a good job explaining science to the public.

### Use of Facebook

The majority (88%) of scientists confirmed having a Facebook account, and 75% indicated that they used Facebook to follow pages that focus on science, while 33% indicated that they were administrators of a page that focuses on science. There was diversity in the science-related activity respondents shared on Facebook. These included sharing their experience in the lab or field (25% indicated that they do so frequently, 52% do so occasionally; from a total of n = 172 scientists who answered this question), finding inspiration for outreach and science communication (24% frequently, 46% occasionally), connecting with other researchers in their field (21% frequently, 37% occasionally), and making corrections to misrepresentations of science (18% frequently, 40% occasionally). When asked to comment further on their use of Facebook as a tool for science communication, 64 respondents provided additional comments. Few of these believed that Facebook provides an effective form of science communication, with just one respondent suggesting that it is “a good way to get out to a lay audience”. Indeed, 7 individuals indicated that Facebook was not effective for science communication, making specific references to the difficulties associated with, in one respondent’s words, “keeping track of the useful responses amongst the banal statements of the obvious or the inflammatory remarks of trolls and anti-science dissenters.” This finding is in accord with the results of a previous study, which found that while an organization’s Facebook page is a good setting for question-answer interactions that allow the public to ask scientists questions about a particular topic, it does not facilitate discussions and presents little opportunity to develop scientific literacy [15].

A large number of scientists (88%) indicated that they regularly use Facebook for personal communication where science is shared with interested friends and family. This is also supported by the literature, which suggests that Facebook is primarily used by the general public to maintain offline relationships [16, 17] and not by users looking to meet or communicate with unknown but like-minded users [18]. Specifically, one respondent in our study stated, “Facebook is not an arena for communicating with scientific peers, but instead for communicating science to interested friends and family, and members of the public” with another stating, “Facebook is a declining resource and other resources such as Pinterest and Twitter will become the prominent social media platforms for engagement with science”.

### Use of Twitter

Although most of the scientists we surveyed were Twitter users, they were relatively new to it. Of the 93% of scientists that identified as having a Twitter account, the majority had had it for less than 2 years. Specifically, 30% had held a Twitter account for 1–2 years, and 29% had an account for less than 1 year, whereas fewer indicated having accounts for longer periods of 2–3, 3–4, or > 5 years (19%, 12% and 7%, respectively). The amount of time that scientists spent on Twitter during a workday was generally between 15–30 minutes (34%), with 28% spending slightly longer (30–60 minutes), and 18% slightly less (< 15 minutes). Few reported spending more than 1 hour (e.g. 13% spend 1–2 hours, 5% spend 2–3 hours, 3% spend 3+ hours). Six individuals reported keeping their Twitter open continuously throughout the day (two of these because they used Twitter frequently for work), whereas a further two reported that they have not used Twitter since they originally signed up for an account.

The majority of scientists (51%) reported that they followed between 101–500 Twitter users themselves, with 15% following slightly more (501–1000) and 19% slightly less (< 100), and very few following more than 1001 (14%). The number of Twitter followers each scientist reported having was typically 101–500 (44%), with 30% reporting having less than 100 followers, and far fewer reporting higher numbers of followers (e.g. 501–1000, 1001–5000 and > 5000, reported by 13%, 12% and 1%, respectively).

**Fig 1:**
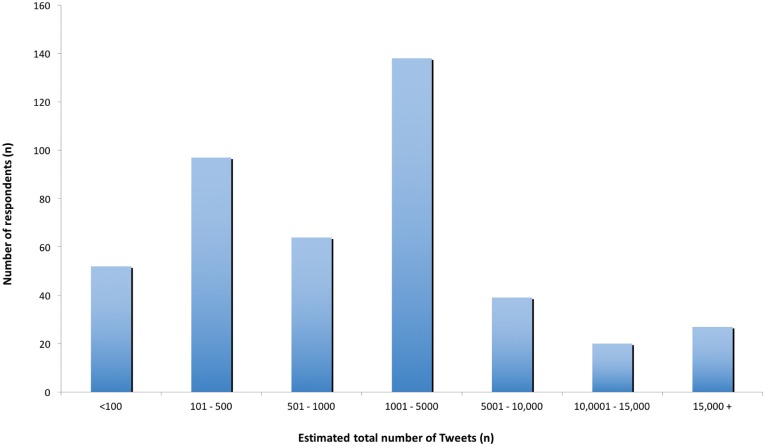
The number of Tweets scientist respondents estimated having posted.

Although it remains debatable what can be considered scientific tweeting, Weller et al. [19] suggest three general requirements. These include: (1) a tweet that includes scientific content, (2) a tweet that is published by a scientist, and (3) a tweet that includes a science-related hashtag. The total number of scientific Tweets scientists estimated that they had posted varied in a slightly bimodal distribution including the highest frequencies at 1001–5000 posts (reported by 32% of scientists) and 101–500 posts (reported by 22%; Fig 1). Estimated individual Tweet posts of 5001–10,000 and <100 were quite similar (9% and 12%, respectively).

Scientists were asked who they aim to connect with using Twitter, by ranking their intended audiences from 1–4 (with 1 being their most preferred). Most indicated “fellow scientists” as their preferred audience (61%), well ahead of “the public” at 31%, or other “organisations” (5%) and “the media” (3%). According to the ranking scale, “scientists” as an audience had an average ranking of 1.6, whereas “the public” averaged a rank of 2.3, “organisations” ranked 2.8 and “the media” ranked 3.3. This pattern is congruent with that reported for academics generally (not specifically in the sciences). Priem & Costello [10] found that scholars in academic institutions predominantly used Twitter to communicate with colleagues and share peer-reviewed literature. It also supports the findings of Bik & Goldstein [20] that scientists are using social media to engage with scholarly communities, although provides slightly less support for their suggestion of scientists using social media as a primary outreach tool to engage the public with their scientific research.

When asked what they Tweet about, respondents ranked a range of different subjects from 1–5 (with 1 being the preferred subject). The most common subject was “research within their own field“, with an average ranking of 2.0. This was followed by “science outreach and communication” (2.7), “personal research” (3.1), “research outside own field” (3.5) and “personal life and experiences” (3.4). Our study also queried the use of Twitter at academic conferences. Most scientists (74%) indicated that they had physically attended conferences that encouraged live Tweeting. Notably, 74% indicated that they had participated in a conference remotely by following Tweets. Of respondents who indicated that they had personally shared Twitter updates from a conference (78%), most stated that they used hashtags created by the conference organisers (64%), while 36% reported using hashtags created by attendees. Respondents estimated that of those people engaging with their Tweets from an academic conference, 57% of interactions were from individuals who were attending the conference and 26% of interactions were from those who were not physically present. This is comparatively different to results presented by Shiffman [21] who found that approximately 90% of tweets from a global conservation biology conference were from individuals not physically attending, but this may vary by discipline (and the associated public interest in that particular discipline).

#### Attitudes towards Twitter

Because our participants were initially targeted from a database of scientists already using Twitter, we took the opportunity to further interrogate scientists’ perceptions about use of this social media platform in particular. Scientists were asked to estimate what proportion of their colleagues used Twitter, and reported a fairly low estimate of 22%. Of those colleagues, however, they estimated that about 24% of their workplace time was spent on Twitter.

When asked to describe their opinions of the top reasons that fellow scientists might be reluctant to use Twitter to communicate science, seven major themes emerged from an analysis of their responses, the most common being a general lack of knowledge of Twitter or “fear of the unknown” (an opinion expressed by 36% of respondents). This included not understanding either how to use Twitter or the point / value of Twitter. Not knowing how to start using social media was also found to be a major impediment for academics using social media in the classroom [22]. The second most common response was a perceived lack of time (proposed by 28% of respondents). Specific comments indicated that scientists were “lacking in time”, or view Twitter as a time-consuming practice or general “waste of time”. This is a sentiment reflected in a survey by Rowlands et al. [23] on the role of social media in the research workflow. Their results also suggest that a lack of time, lack of clarity of the exact benefits of social media, and general uncertainty act as a barrier to social media use in the workplace. Of note, time constraints is also cited as the biggest obstacle to outreach for scientists [24]. Further linked themes emerged around the suggestion that Twitter is “silly or frivolous” (8% of responses) and that it “lacks scientific content” and is not a scientifically “rigorous enough media” to support professional scientific debate—often with reference to peer review (a view represented in 3% of responses). Interestingly, survey participants in the Rowlands et al. study [23] held an opposing view, suggesting that they trusted their own ability to evaluate whether information was trustworthy, that their online networks would filter “rubbish” and that inclusion of varied forms of authority, other than peer-review, were beneficial. A fourth theme attributed reluctant use of Twitter to a lack of privacy (6%), and a fifth to a “lack of characters” or dislike of the format (10%). Less frequent responses included aversions to the content being shared on (n = 4), it being unprofessional (n = 3) and it being age-biased (n = 2).

Participants were also asked to provide their opinions of the top benefits of using Twitter to communicate science. Their answers could be grouped in five major themes. The most common referred to the size and diversity of the potential audience, i.e. members of the public using Twitter (28%). Another frequently noted benefit was the ease of communicating snippets, referring specifically to the size of tweets, the short amount of time it takes, and the accessibility of it (proposed by 26% of respondents). A further benefit emerged in reference to networking and collaborating with other scientists (proposed by 20%). Other respondents attributed benefits of their use of Twitter to the content they were able to access and share (14%) and the ability to communicate their science directly with the public (7%). The remaining 2% (n = 8) of responses were categorized as ‘other’. These suggested that using Twitter to communicate science is ‘fun’ (n = 2), helped hone communication skills (n = 2), has users that are open and more intimate (n = 2), and acts as a way to access science journalists (n = 2).

Finally, scientists were asked to report on their perceptions of workplace policy on social media use. Most scientists reported being not aware of any policy (44%), while 36% said definitively that there was no such policy and only 20% reported that they knew their workplace had a policy. For those institutions with a social media policy, respondents indicated that there was no clear and generalised message being communicated internally about what scientists were allowed to share on Twitter. Any regulations noted appeared to consist only of requests that scientists using Twitter consider the institution’s reputation, limit the amount of time spent at work using social media, and/or avoid giving comment on scientific issues that are outside of an individual’s area of expertise.

## Conclusion

Our results demonstrate that while social media usage has yet to be widely adopted, scientists in a variety of disciplines use these tools to exchange scientific knowledge. While many scientists followed science-themed Facebook pages, most suggested that they use it only for personal communication where science is shared with interested colleagues, family and friends. Few believed that Facebook is suitable for science communication to the general public. Similarly, a high percentage of scientists read science blogs, and approximately half had written their own science blog. Many shared science-themed blogs with their professional colleagues and most believed that blogs have a role to play in increasing public understanding of science. Scientists using Twitter appears to be a new movement, with many participants suggesting they have had their account for less than two years. Many scientists used Twitter to communicate specifically with other scientists. Some used it as a forum to share their research directly with the public and media, however, most saw it as a tool to share research within their field and to stay updated with science outreach and communication activities. Most scientists had attended conferences that encouraged live Tweeting and many had followed a conference remotely through Tweets. The most common barriers to Twitter perceived by scientists were a lack of time and a lack of knowledge. A few comments were made around a lack of scientific validity and similarly, that Twitter is “silly or frivolous” but the general consensus was that scientists do not have enough time to use Twitter. By comparison, the most common perceived benefits of Twitter were the size and diversity of the audience reached by Twitter and the ability to network with other scientists. Others mentioned the ability to engage with the public, but it was mostly focused around expanding knowledge and networks. Very few respondents use academia-themed social networks, such as Academia.edu or Mendeley.

The results of this study add to our general understanding of the use of social media by academic scientists. Despite the professional benefits associated with social media use, relatively few academic scientists currently use these tools. Misunderstandings of the disadvantages of social media use may contribute to their relatively limited use, which could be corrected by professional development training workshops or clearer departmental social media usage policies. This could help ensure that more scientists would enjoy the professional benefits of social media use. Our sample of scientists, targeting those already using social media (and in particular Twitter), was ultimately biased towards a younger demographic, predominantly in the life sciences. Although our sampling methods and survey questions were fairly simplistic, they have provided a solid baseline from which to compare future trends (e.g. a point of comparison with other academic science disciplines, age groups, or cultures) and develop more nuanced investigations of the affordances and constraints of communication technology.

## Supporting Information

S1 File Scientists & Social Media Survey. Supporting information detailing questions included in the survey.
(PDF)
