# Supplementary material for: How Are Scientists Using Social Media in the Workplace?
Source: PLoS One. 2016 Oct 12;11(10):e0162680. doi: 10.1371/journal.pone.0162680 (PMC5061391; doi:10.1371/journal.pone.0162680)
Supplement: S1 File — Supporting information detailing questions included in the survey. (PDF) [file pone.0162680.s001.pdf]

Scientists & Social Media Survey

INFORMATION SHEET FOR PARTICIPANTS

Thank you for showing an interest in this project. Please read this information sheet carefully before deciding whether or not to participate. If you decide to participate we thank you. If you decide not to take part there will be no disadvantage to you and we thank you for considering our request.

What is the Aim of the Project?

The project is being undertaken as part of a Masters of Science Communication at the Centre for Science Communication, at The University of Otago. The main aim of the project is to examine current attitudes held by scientists towards the social media platform, Twitter.

What Type of Participants are being sought?

Scientists who use social media.

What will Participants be Asked to Do?

Should you agree to take part in this project, you will be asked to answer a 5-10 minute online survey on attitudes towards social media and provide simple demographic information to be used in statistical analysis. Your answers will remain completely anonymous.

What Data or Information will be Collected and What Use will be Made of it?

Some written responses may be quoted in a Masters thesis and other publications, but no identifiers will be used. The data collected will be securely stored in such a way that only those involved in this research project will be able to gain access to it. At the end of the project any raw data on which the results of the project depend will be retained in secure storage for five years, after which it will be destroyed. The results of the project may be published and will be available in the University of Otago Library (Dunedin, New Zealand), but all data will remain anonymous.

Can Participants Change their Mind and Withdraw from the Project?

You may withdraw from participation in the survey at any time and without any disadvantage to yourself of any kind.

By clicking on the “Next” button below, you indicate that you have agreed to participate in this project. If you do not agree, please close this page.

CONTACT DETAILS

Q1. Full Name

Q2. Email Address

Please note that all contact details will remain anonymous. They will be destroyed following the completion of this study.

DEMOGRAPHICS

Q3. Location (Country)

Q4. Gender

Male

Female

Other (please specify)

Q5. Age

18 – 20

21 – 29

30 – 39

40 – 49

50 – 59

60 or older

Q6. Highest Level of Education Completed

High School

Bachelors Degree

Honours Degree

Post-Graduate Diploma

Masters Degree

Doctor of Philosophy

Other (please specify)

Q7. Current Institution (if applicable)

Q8. Field or Area of Expertise

Anatomy

Anthropology

Applied Sciences

Archaeology

Astronomy

Biochemistry

Biology

Biotechnology

Botany

Chemistry

Conservation Biology

Earth Science

Ecology

Energy Science

Environmental Science

Epidemiology

Fishery Science

Forestry

Genetics

Geography

Geology

Hydrology

Laboratory Science

Marine Science

Medicine

Meteorology

Microbiology

Microscopy

Molecular Biology

Nanoscience

Neurology

Neuroscience

Paleontology

Pathology

Pharmacology

Physics

Physiology

Psychology

Science Communication

Science Education

Seismology

Systematics

Viticulture

Volcanology

Zoology

Other (please specify)

GENERAL USE OF SOCIAL MEDIA

Q9. Which of the following social media services do you use?

Facebook	Research Gate	FourSquare
Twitter	Pinterest	Wordpress
Google +	Instagram	Blogger
LinkedIn	Tumblr	Mendeley
MySpace	Reddit	Other (please specify)

Q10. What social media service do you use most often (rank with 1 being the most often)?

This question requires you to produce a ranked list.

When you make your first selection, the other options will be sorted automatically by number below your first option, so please adjust these numbers to suit. The final result should be a list in order of importance.

Facebook	Research Gate	FourSquare
Twitter	Pinterest	Wordpress
Google +	Instagram	Blogger
LinkedIn	Tumblr	Mendeley
MySpace	Reddit	

USE OF FACEBOOK

Q11. Do you have a personal Facebook account?

Yes

No

Q12. Have you "liked" any Facebook pages that focus on science?

Yes

No

Q13. Are you the administrator of any Facebook pages?

Yes (if yes, continue to page 7)

No (if no, continue to page 8)

Q14. What is the subject of the page/s you are an administrator for? (select multiple)

Anatomy
Anthropology
Applied Sciences
Archaeology
Astronomy
Biochemistry
Biology
Biotechnology
Botany
Chemistry
Conservation Biology
Earth Science
Ecology
Energy Science
Environmental Science
Epidemiology
Fishery Science
Forestry
Genetics
Geography
Geology
Hydrology
Laboratory Science
Marine Science
Medicine
Meteorology
Microbiology
Microscopy
Molecular Biology
Nanoscience
Neurology
Neuroscience
Paleontology
Pathology
Pharmacology
Physics
Physiology
Psychology
Science Communication
Science Education
Seismology
Systematics
Viticulture
Volcanology
Zoology
Other (please specify)

Q15. You have used Facebook to (select multiple):

Find inspiration for outreach and science

Access general scientific resources

Access peer-reviewed papers

Receive feedback on an academic paper

Collaborate with another researcher in your field

Find inspiration for an academic paper or similar project

Share your experience in the lab or field

Connect with other researchers in your field

Make corrections to misrepresentations of science

Share peer-reviewed papers

Give feedback on an academic paper

Other (please specify)

Q16. Do you have any further comments about using Facebook to communicate science?

USE OF BLOGS

Q17. Do you read science blogs?

Yes (if yes, continue to page 12)

No (if no, continue to page 11)

Q18. Do you think that science blogs do a good job explaining science to the public?

Yes

No

Other (please specify)

Q19. Have you ever shared a post from a science blog with professional colleagues?

Yes

No

Other (please specify)

Q20. Have you ever written for a science blog?

Yes

No

Other (please specify)

Q21. If you do not read science blogs, why not?

Q22. What are your 3 favourite science blogs?

Q23. Do you have any further comments about using blogs to communicate science?

TWITTER USE

Q24. Do you have a Twitter account?

Yes (if no, continue to page 15)

No (if no, continue to page 24)

Q25. What is your Twitter username?

Q26. Approximately how long have you been a member of Twitter?

< 1 year

2 years

3 years

4 years

5 years

6 years

7 years

8 years

Q27. How many followers do you have?

This information can be found on your Twitter profile.

Q28. How many users are you following?

This information can be found on your Twitter profile.

Q29. How many Tweets have you posted?

This information can be found on your Twitter profile.

Q30. In a typical day, about how much time do you spend using Twitter?

Less than 15 minutes

15 - 30 minutes

30 minutes - 1 hour

1 hour - 2 hours

2 hours - 3 hours

3 hours - 4 hours

More than 4 hours

Other (please specify)

Q31. Who do you aim to connect with using Twitter? (please rank with 1 being the most desired)

Public

Scientists

Media

Scientific Organisations

Q32. What do you Tweet about? (please rank with 1 being the most desired)

Personal life & experiences

Research OUTSIDE own field or area of expertise

Science outreach & communication

Personal research

Research WITHIN own field or area of expertise

Q33. Other than the options given in the question above, what do you Tweet about?

Q34. You have used Twitter to:

Find inspiration for outreach and science

Access general scientific resources

Access peer-reviewed papers

Receive feedback on an academic paper

Collaborate with another researcher in your field

Find inspiration for an academic paper or similar project

Share your experience in the lab or field

Connect with other researchers in your field

Make corrections to misrepresentations of science

Share peer-reviewed papers

Give feedback on an academic paper

Other (please specify)

Q35. If you have GIVEN feedback on an academic paper through Twitter, was it:

Within your area of expertise

Outside of your area of expertise

Q36. If you have RECEIVED feedback on an academic paper through Twitter, was it:

Within your area of expertise

Outside of your area of expertise

Q37. If you have collaborated with another researcher through Twitter, please elaborate on your experience.

Q38. To the best of your knowledge, what percentage of individuals within your institution or workplace actively use Twitter?

10% 20% 30% 40% 50% 60% 70% 80% 90% 100% None

Q39. To the best of your knowledge, what percentage of individuals within your department actively use Twitter?

10% 20% 30% 40% 50% 60% 70% 80% 90% 100% None

Q40. Has your institution or workplace implemented a policy on social media use?

Yes (if yes, continue to page 21)

No (if no, continue to page 22)

Don't Know

Q41. What is the general theme or message of you institution or workplace's policy on social media?

Q42. Have you ever attended an academic conference where Tweeting was encouraged?

Yes

No

Q43. Have you ever Tweeted from an academic conference using a hashtag?

Yes (if yes, go to page 23)

No (if no, go to page 24)

Q44. Have you ever attended an academic conference remotely by following a hashtag?

Yes

No

Q45. Was the hashtag you used:

Set by the conference organisers

Created by attendees

Q46. To the best of your knowledge, what percentage of the interactions with your Tweets from the conference were from individuals NOT attending?

10% 20% 30% 40% 50% 60% 70% 80% 90% 100% None

Q47. To the best of your knowledge, what percentage of the interactions with your Tweets from the conference were from individuals ATTENDING?

10% 20% 30% 40% 50% 60% 70% 80% 90% 100% None

Q48. In your opinion, what are the top 3 reasons why scientists may be reluctant to communicate science?

Q49. In your opinion, what are the top 3 benefits of using Twitter for science communication?

Q50. In your opinion, what are the top 3 disadvantages of using Twitter?

Q51. What are the top 3 most common opinions of Twitter held by other researchers you have spoken to?

Q52. Do you have any further comments about using Twitter to communicate science?
